# Novel compound heterozygous mutations in plasminogen (p.Gly568Arg/p.Ala620Thr) impair protein structure and function in type II deficiency: mechanistic insights into a hereditary thrombogenic disorder

**DOI:** 10.1186/s13023-025-04122-3

**Published:** 2025-12-15

**Authors:** Yifan Lu, Fengjiao Wang, Dandan Yu, Haixiao Xie, Yanhui Jin, Mingshan Wang, Lihong Yang

**Affiliations:** Department of Clinical Laboratory, Key Laboratory of Clinical Laboratory Diagnosis and Translational Research of Zhejiang Province, The First Affiliated Hospital of Wenzhou Medical University, Wenzhou, Zhejiang China

**Keywords:** PLG, Hereditary PLG deficiency, In vitro expression, Thrombus formation

## Abstract

**Background:**

Hereditary plasminogen (PLG) deficiency represents an extremely rare autosomal recessive disorder characterized by impaired fibrinolytic capacity resulting from diminished PLG enzymatic activity. In this study, we identify and characterize a novel compound heterozygous PLG mutation clinically associated with cerebral infarction. Our findings demonstrate that structural conformational alterations in the mutation PLG protein disrupt normal fibrinolytic pathway function.

**Methods:**

In this study, the proband presented at the First Affiliated Hospital of Wenzhou Medical University with a chief complaint of “left-sided limb weakness lasting for two days.” PLG activity (PLG: A) and PLG antigen (PLG: Ag) levels were measured in the proband and eight family members spanning three generations using the chromogenic substrate assay(CSA) and enzyme-linked immunosorbent assay (ELISA), respectively. The genetic mutation site was identified through direct DNA sequencing. Bioinformatics software was employed to analyze the conservation and potential pathogenicity of the mutation site. A mutation protein model was constructed to investigate structural alterations in the protein before and after the mutation. Furthermore, a recombinant plasmid expression vector was developed, and the in vitro expression of the recombinant PLG protein was evaluated using real-time quantitative PCR (RT-qPCR), ELISA, and Western blot(WB) analysis.

**Results:**

The propositus had a significantly reduced PLG: A to 27%, but the PLG: Ag level was normal at 103%, and was diagnosed with type II plasminogen deficiency (dysplasminogenemia). Sanger sequencing identified compound heterozygous missense mutations in the PLG gene: c.1702G > A (p.Gly568Arg) in exon 14 and c.1858G > A (p.Ala620Thr) in exon 15. Both mutation sites are highly conserved across species, and four independent bioinformatics tools consistently predicted pathogenic effects. Molecular modeling demonstrated that the p.Gly568Arg substitution causes steric hindrance through side chain elongation and establishes a new hydrogen bond with Leu686; p.Ala620Thr destabilized the catalytic triad (His603-Asp646-Ser741). In vitro functional assays, including RT-qPCR, ELISA, and WB analysis, confirmed that neither mutation significantly altered the expression level of mRNA、protein biosynthesis and secretion. However, the PLG: A/PLG: Ag ratio in the media was markedly reduced compared to wild-type controls.

**Conclusions:**

We report the first case worldwide of a novel p.Gly568Arg mutation in the PLG gene, which coexists with the p.Ala620Thr mutation in a compound heterozygous form. Structural and functional analyses indicate that although the transcription, protein synthesis and secretion of the PLG gene are not affected, these two mutations specifically impair the catalytic activity of the PLG protein through different conformational changes. This expands the mutation spectrum and clarifies a novel thrombosis mechanism in type II PLG deficiency.

**Supplementary Information:**

The online version contains supplementary material available at 10.1186/s13023-025-04122-3.

## Introduction

The fibrinolytic system serves as a crucial regulatory network for vascular homeostasis, primarily through fibrin degradation to prevent pathological thrombosis [1]. This system comprises plasminogen (PLG), its activators (tissue-type PLG activator [t-PA] and urokinase-type PLG activator [u-PA]), inhibitors and α2-antiplasmin), and fibrin substrates - collectively forming a precisely balanced feedback loop [2, 3]. PLG functions as the fibrinolytic system’s core zymogen, tPA/uPA-mediated activation converts PLG to plasmin, which cleaves fibrin and extracellular matrix(ECM) proteins [4]. Furthermore, plasmin proteolytically processes inflammatory mediators, thereby modulating immune responses [5], and promotes epithelial migration via specific receptor interactions, which facilitates wound repair [6].

Hereditary PLG deficiency comprises a spectrum of disorders caused by pathogenic PLG gene mutations, characterized by either quantitative deficiency or dysfunctional PLG protein. This impairment disrupts fibrinolytic homeostasis, resulting in a rare prothrombotic state with an estimated prevalence of 1.6 cases per million individuals [7]. The PLG gene comprises 19 exons and 18 introns [8]. The precursor protein encoded by this gene consists of 810 amino acids and ultimately gives rise to the mature PLG protein. Structurally, PLG consists of three primary domains: an N-terminal peptide (NTP), five kringle domains (K1-K5), and a C-terminal serine protease (SP) domain [9]. PLG specifically binds to fibrin via its kringle domains. The Arg561-Val562 site within the SP domain functions as a critical site for proteolytic activation. Following cleavage at this site, PLG is activated into catalytically active plasmin, which subsequently mediates efficient fibrin degradation [10]. Current evidence indicates that pathogenic mutations are mainly concentrated in the Kringles domain and the SP domain. Mutations in these specific regions lead to different clinical phenotypes. Mutations in the Kringles domain, such as p.Lys38Glu, usually result in local fibrin deposition, presenting as ligneous conjunctivitis. Mutations in the SP domain, such as p.Arg561His, impair systemic fibrinolysis, leading to thrombotic events or organ fibrosis [8, 11].

We conducted an integrated genetic and functional analysis of PLG mutations in a proband presenting with ischemic stroke and reduced PLG: A activity, along with affected family members.This study elucidated the possible molecular mechanisms underlying PLG deficiency and provides a foundation for refining diagnostic approaches and developing targeted therapeutic strategies.

## Materials and methods

### Clinical evaluation and coagulation profiling

We collected clinical data from the proband and family members. Venous blood was anticoagulated with 3.2% sodium citrate (9:1 v/v) and processed within 2 h. Following centrifugation (1,500×g, 15 min, 22℃), platelet-poor plasma (PPP) was aliquoted and stored at -80℃ pending analysis. The buffy coat was isolated for genomic DNA extraction. PLG: A, protein C activity (PC: A), and antithrombin III activity (AT: A) were measured using chromogenic substrate assay(CSA) on a STA-R-Max analyzer (Diagnostica Stago, France). Protein S activity (PS: A) was determined by clotting assay on the same platform, while PLG antigen (PLG: Ag) levels were quantified using a commercial enzyme-linked immunosorbent assay (ELISA) kit (Jianglai Biotechnology, China). All analyses are performed in duplicate following both Clinical and Laboratory Standards Institute (CLSI) guidelines and the manufacturer’s protocols.

### Gene analysis

Genomic DNA was extracted from peripheral blood leukocytes using the TIANamp Genomic DNA Kit (DP304; Tiangen Biotech, Beijing, China). For PLG gene analysis (Gene ID: 5340; RefSeq NM_000301.3), we designed 19 primer pairs encompassing all exons and flanking splice junctions (± 50 bp) according to published methodology [12]. Polymerase chain reaction (PCR) amplification was performed under optimized conditions, with subsequent bidirectional Sanger sequencing using an ABI 3730xl Genetic Analyzer (Applied Biosystems). All primers (HPLC-purified) were commercially synthesized (Shanghai Saiheng Biotechnology). Sequence traces were analyzed with Chromas Pro (Technelysium) and aligned to the PLG reference sequence (NCBI RefSeq NG_016200.1) via Primer-BLAST (https://www.ncbi.nlm.nih.gov/tools/primer-blast/). Mutations identified in the proband were classified according to American College of Medical Genetics and Genomics(ACMG) guidelines and verified in family members through targeted sequencing.

### In Silico analysis

Pathogenicity predictions for identified mutations was performed using six established prediction algorithms: MutationTaster (https://www.mutationtaster.org/), SIFT (https://sift.bii.a-star.edu.sg/), PROVEAN (https://provean.jcvi.org/), FATHMM (https://fathmm.biocompute.org.uk/), CADD (https://cadd.gs.washington.edu/score) and REVEL (https://asia.ensembl.org/Tools/VEP). Evolutionary conservation was assessed via Clustal X 2.1 multiple sequence alignment of homologous RefSeq sequences (https://www.ncbi.nlm.nih.gov/nuccore/). PyMOL (v2.7) modeling revealed mutation-specific structural changes. Furthermore, the population-based allele frequencies for both mutations were obtained from the gnomAD database.

### Construction of recombinant PLG expression vector

The pCDH-copGFP-T2A-Puro plasmid (System Biosciences, USA) was used for PLG overexpression. Primers were designed based on the full-length PLG complete coding sequence (CDS), and the PCR product was gel-purified, digested with XbaI/NotI (Takara Bio, Japan), and ligated into the linearized vector. Site-directed mutagenesis was performed using the Mut Express II Fast Mutagenesis Kit V2 (Vazyme) with custom-designed primers. All constructs (wild-type and mutation) were verified by bidirectional Sanger sequencing. Human embryonic kidney 293T (HEK293T) cells were cultured in Dulbecco’s Modified Eagle Medium (DMEM, Gibco) supplemented with 10% heat-inactivated fetal bovine serum (FBS, Invitrogen), 100 U/ml penicillin, and 100 µg/ml streptomycin (Beyotime) under standard conditions. For transfection, cells at 70–80% confluency were transfected with plasmid constructs using Lipofectamine 5000 (Biodai Biotechnology, China) according to the manufacturer’s protocol. After 48 h, cells and conditioned medium were collected. The conditioned medium was concentrated using Amicon Ultra-30 K centrifugal filters (Millipore; 4,000 ×g, 30 min).

### Total RNA extraction and quantitative real-time PCR (RT-qPCR)

Total RNA was extracted from transfected cells using RNAiso Plus (Takara, Japan). Complementary DNA (cDNA) was synthesized with HiScript II Q RT SuperMix (Vazyme), followed by RT-qPCR using Taq Pro Universal SYBR qPCR Master Mix (Vazyme) on a QuantStudio™ 5 system (Thermo Fisher Scientific). cDNA)was synthesized with HiScript II Q RT SuperMix(Vazyme, China) on a QuantStudio™ 5 system (Thermo Fisher Scientific, America). GAPDH served as the endogenous control. Wild-type and mutation PLG mRNA expression levels were quantified using the comparative Ct (2 − ΔΔCt) method (Supplementary Table 1).

### Western blot (WB) and enzyme-linked immunosorbent assay (ELISA)

The plasmid was transfected into the medium containing 10% fetal bovine serum and cultured for 48 h. The cells and media were collected for analysis. Cell proteins were extracted using RadioImmunoPrecipitation Assay (RIPA) Buffer lysis buffer supplemented with phenylmethylsulfonyl fluoride (PMSF), while supernatants were concentrated by centrifugal ultrafiltration. Proteins were denatured, separated by 10% sdium dodecyl sulfate-polyacrylamide gel electrophoresis (SDS-PAGE), and transferred to polyvinylidene fluoride (PVDF) membranes. After blocking with 5% skim milk in Tris-buffered saline with Tween-20 (TBST) for 2 h at room temperature, membranes were incubated overnight at 4 °C with primary antibodies: anti-PLG (1:2000; Proteintech, China) and anti-GAPDH (1:2000; Proteintech, China). Following washes, membranes were incubated with HRP-conjugated goat anti-rabbit IgG (1:5000; Beyotime, China) for 1 h at room temperature. Protein bands were visualized using an enhanced chemiluminescence (ECL) detection kit (Yasen Biotechnology) and quantified by densitometry. The protein bands were visualized using an ECL ultra-sensitive detection kit (Yasen Biotechnology, China) and quantified by densitometry. PLG: Ag levels in cell lysates and supernatants were quantified by ELISA (Jianglai Biotechnology, China). PLG: A in supernatants was measured using chromogenic substrate assays on a STA-R Max analyzer. Specific activity was calculated as the PLG: A/PLG: Ag ratio and normalized to wild-type PLG (100%).

## Results

### Clinical investigation, laboratory findings, and PLG mutations

**Fig. 1:**
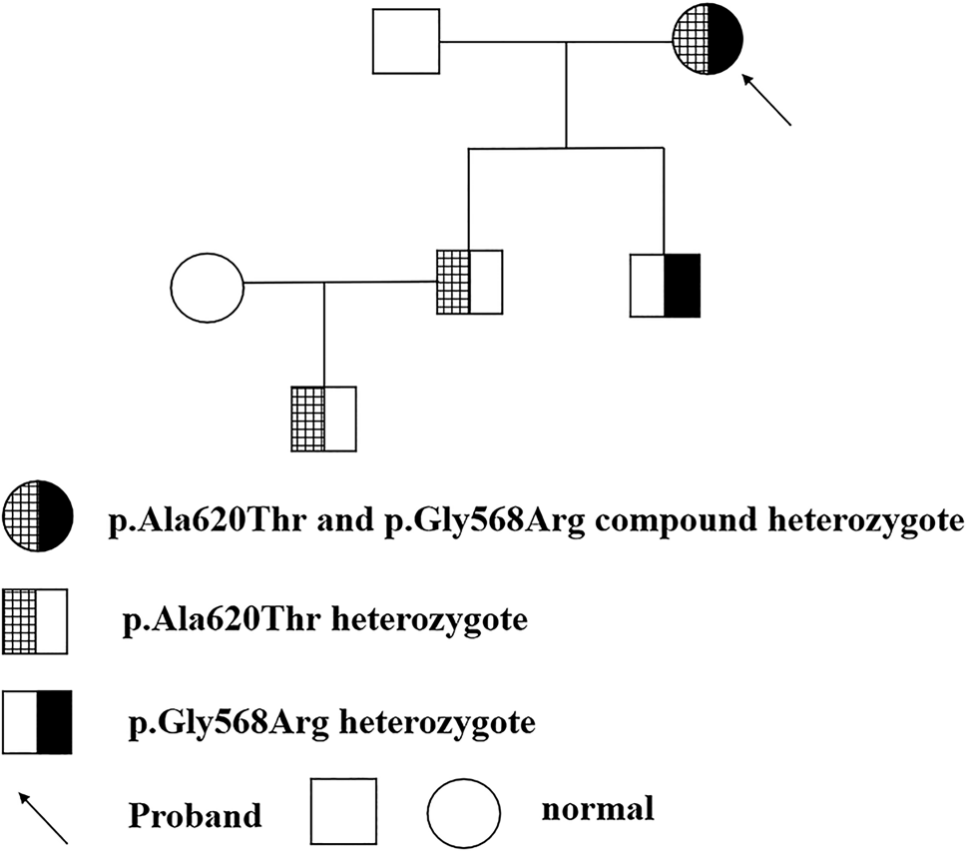
Pedigree of the PLG-deficient family

**Fig. 2:**
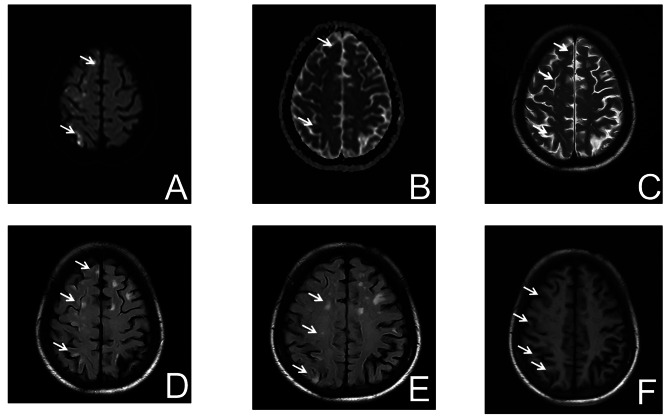
Brain MRI findings. **A**: DWI head MRI; **B**: ADC head MRI; **C**: T2-weighted head MRI; **D**: Flair head MRI; **E **,**F**: T1-weighted head MRI

**Fig. 3:**
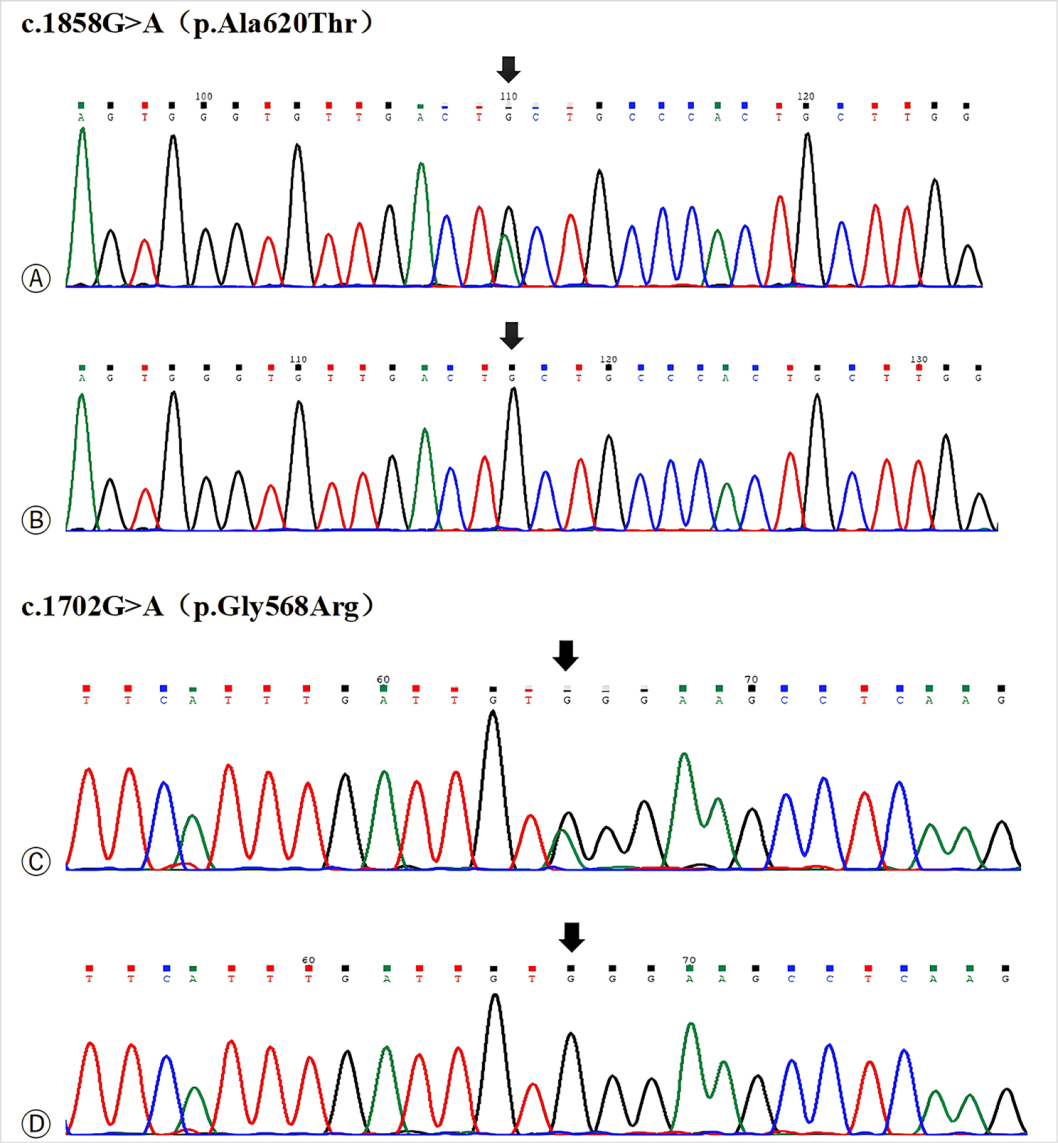
Direct sequencing results of the proband’s genes. Ⓐ: Direct sequencing of the c.1858G > A mutant type. Ⓑ: Direct sequencing of the c.1858G > A wild type. Ⓒ: Direct sequencing of the c.1702G > A mutant type. Ⓓ: Direct sequencing of the c.1702G > A wild type. The black arrows indicate the mutation sites

This study examined an eight-member, three-generation consanguineous family (Fig. 1). The 60-year-old female proband presented to our institution with two-day persistent left hemiplegia. Brain Magnetic resonance imaging(MRI) demonstrated multiple acute ischemic infarcts involving the right frontal, parietal, temporal, occipital, and insular lobes (Fig. 2). Thromboelastography (TEG) revealed a hypercoagulable state (Supplementary Fig. 1), prompting treatment with dual antiplatelet therapy, atorvastatin for dyslipidemia, and neuroprotective butylphthalide, which stabilized her condition. Coagulation studies showed markedly reduced PLG: A (27%) in the proband despite normal antigen levels. Her two sons and grandson exhibited approximately 50% of normal PLG: A with preserved antigen levels, while other family members showed normal coagulation parameters.After comprehensive screening ruled out common thrombotic causes, targeted genetic analysis was pursued, which identified two heterozygous missense mutations in the proband’s PLG gene: c.1702G > A (p.Gly568Arg) in exon 14 and c.1858G > A (p.Ala620Thr) in exon 15 (Fig. 3). Segregation analysis revealed each son and grandson carried one mutation (either p.Gly568Arg or p.Ala620Thr). All unaffected family members had wild-type genotypes (Table 1).

**Table 1:** Clinical and coagulation profiles in a PLG-deficient family

Family members	PLG: A(%)	PLG: Ag(%)	PC: A(%)	PS: A(%)	AT: A(%)	Mutation type	Clinical manifestations
Proband(I1)	27	103	106	97	99	p.Ala620Thr p.Gly568Arg	Acute Ischemic Stroke
Husband(I2)	93	120	111	133	103	Wild type	asymptomatic
eldest son’s wife(Ⅱ1)	107	123	93	129	115	Wild type	asymptomatic
Eldest son(Ⅱ2)	54	116	107	104	107	p.Ala620Thr	asymptomatic
Youngest son(Ⅱ3)	42	104	97	113	105	p.Gly568Arg	asymptomatic
Grandson(Ⅲ1)	55	119	95	104	109	p.Ala620Thr	asymptomatic
Reference range	75 ~ 140	50 ~ 150	70 ~ 130	65 ~ 135	96~ 118	/	/

PLG: A: plasminogen activity; PLG: Ag: plasminogen antigen; PC: A: protein C activity; PS: A: protein S activity; AT: A: antithrombin activity

### In silico analysis

**Fig. 4:**
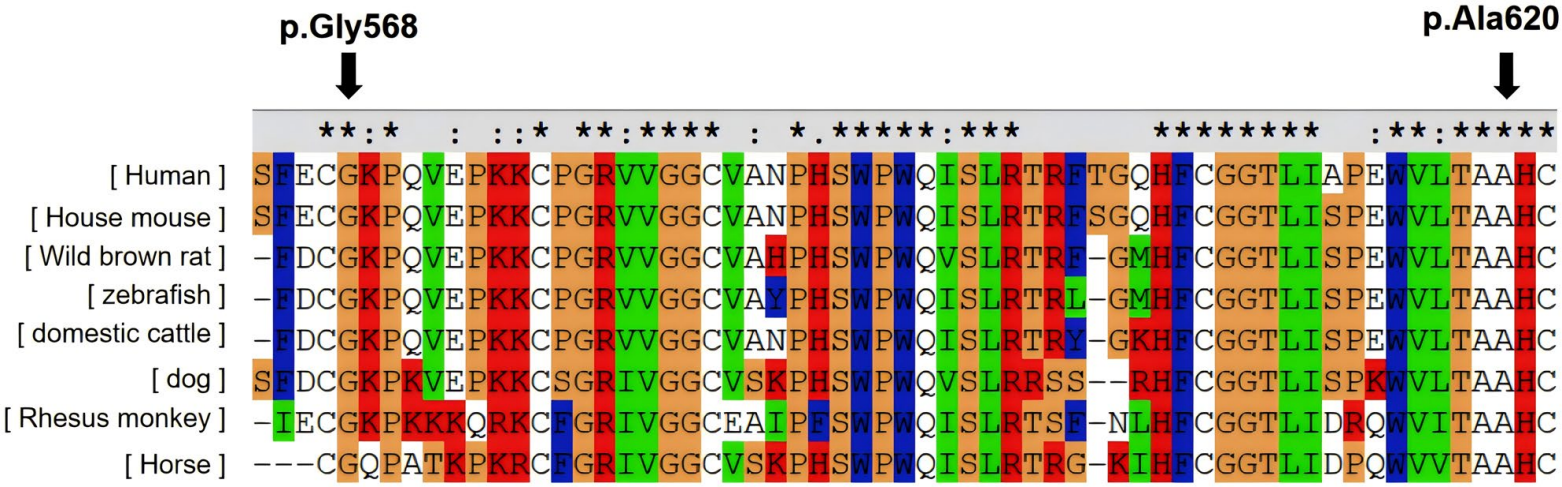
Conservation analysis of p.Gly568Arg and p.Ala620Thr across different species.(★, ∶, and · indicate that the amino acid site is completely conserved, highly conserved, and weakly conserved, respectively)

**Fig. 5:**
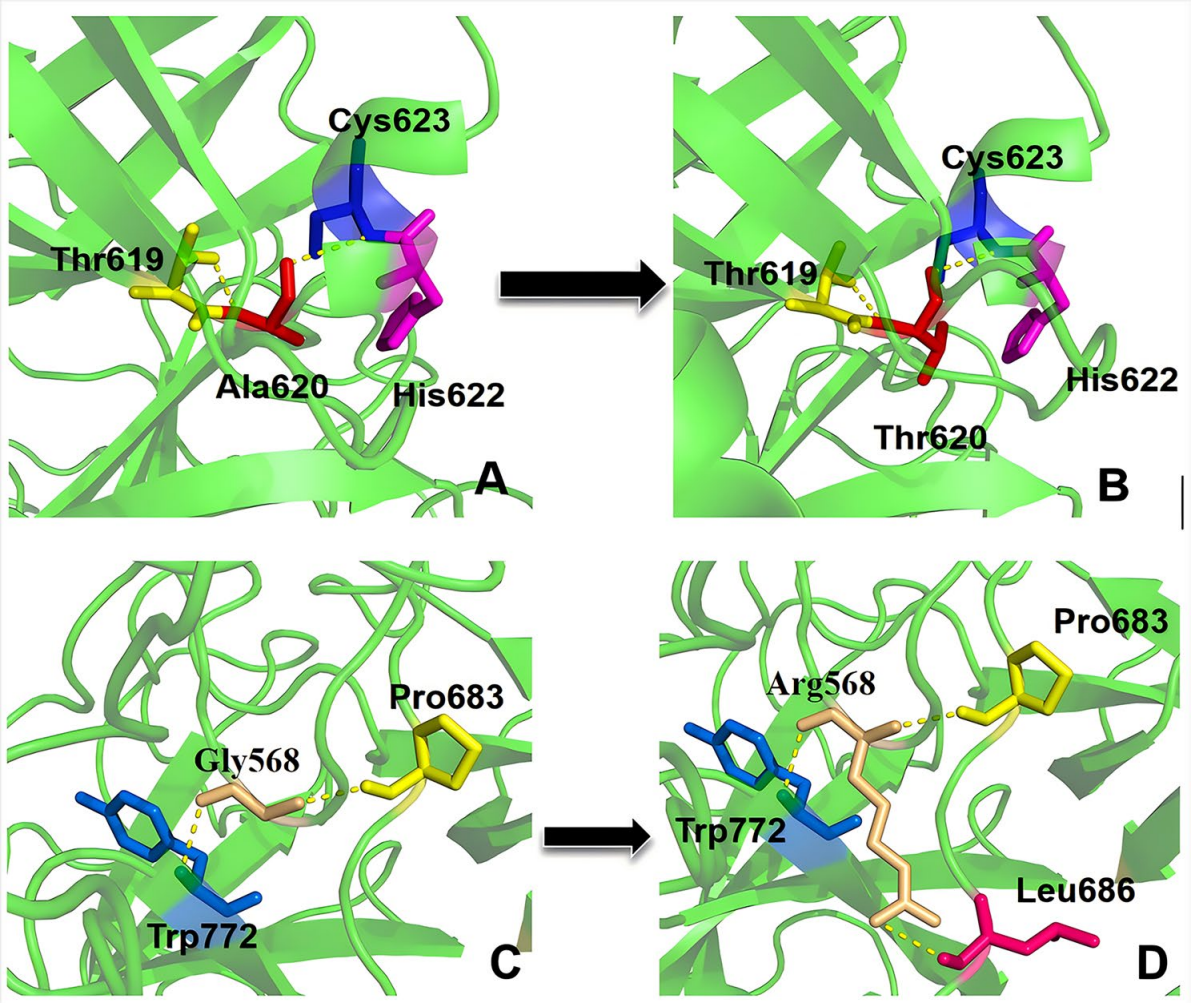
Protein spatial model structure diagrams of p.Gly568Arg and p.Ala620Thr. (**A**) The p.Ala620Thr wild-type PLG protein molecular model; (**B**) The p.Ala620Thr mutant PLG protein molecular model; (**C**) The p.Gly568Arg wild-type PLG protein molecular model; (D) The p.Gly568Arg mutant PLG protein molecular model

Six computational prediction tools (SIFT, PolyPhen-2, MutationTaster, PROVEAN, CADD, REVEL) consistently classified both p.Gly568Arg and p.Ala620Thr mutations as pathogenic (Table 2). Cross-species alignment demonstrated complete evolutionary conservation of both residues across seven representative species (Fig. 4). Structural modeling indicated that the p.Gly568Arg disrupted the Gly568-Trp772-Pro683 hydrogen bond network while forming a new interaction with Leu686; the p.Ala620Thr introduced steric constraints through threonine side-chain extension(Fig. 5). The Ala620Thr variant was observed at an allele frequency of 0.0509% [gnomAD v4.1.0 (GRCh38)], in contrast to the Gly568Arg variant, which is novel (absent from gnomAD).

**Table 2:** Pathogenicity prediction of genetic mutations

Mutation	MutationTaster	SIFT	PROVEAN	FATHMM	CADD	REVEL
p.Gly568Arg	1.000	0.002	-5.85	-1.85	28.9	0.690
p.Ala620Thr	1.000	0.088	-2.77	-3.76	29.5	0.797

Note: The prediction value range of MutationTaster is 0 to 1, and the closer to 1, the more likely it is to be pathogenic; the score range of SIFT is 0 to 1, and a score < 0.05 is predicted to be deleterious; the predicted result score range of PROVEAN is -14 to 14, with a threshold of -2.5, and a score of -14 to 2.5 is predicted to be deleterious, and the smaller the score, the more deleterious it is; a score < 0 of FATHMM is predicted to be deleterious; A CADD score of ≥ 10 suggests potential harm, ≥ 20 (top 1%) indicates likely harm, and ≥ 30 (top 0.1%) indicates highly likely harm. A REVEL score > 0.75 is highly likely to be pathogenic, 0.6–0.75 is likely to be pathogenic; 0.4–0.6 is undetermined and requires combination with other methods; <0.4 is inclined to be benign, 0.15–0.4 is likely to be benign, and < 0.15 is highly likely to be benign

### In vitro expression study of transfected HEK cells

**Fig. 6:**
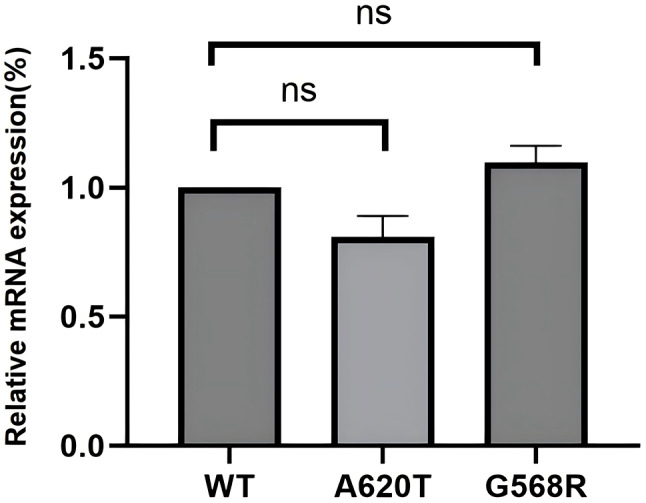
The relative expression levels of mRNA of wild-type and mutant recombinant plasmids (“ns” indicates no significant difference, *p* > 0.05)

**Fig. 7:**
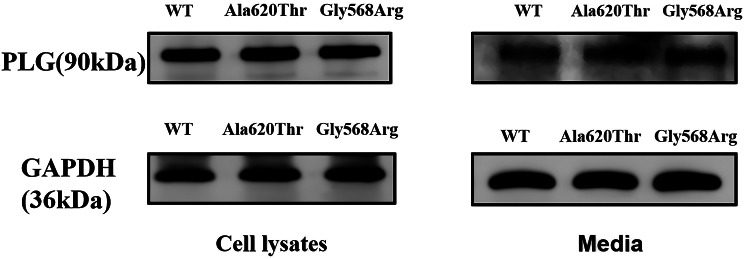
The WB experimental protein expression results of PLG protein in HEK293T cells. (The upper band is the target protein band, and the lower band is the internal reference protein GAPDH band.)

**Fig. 8:**
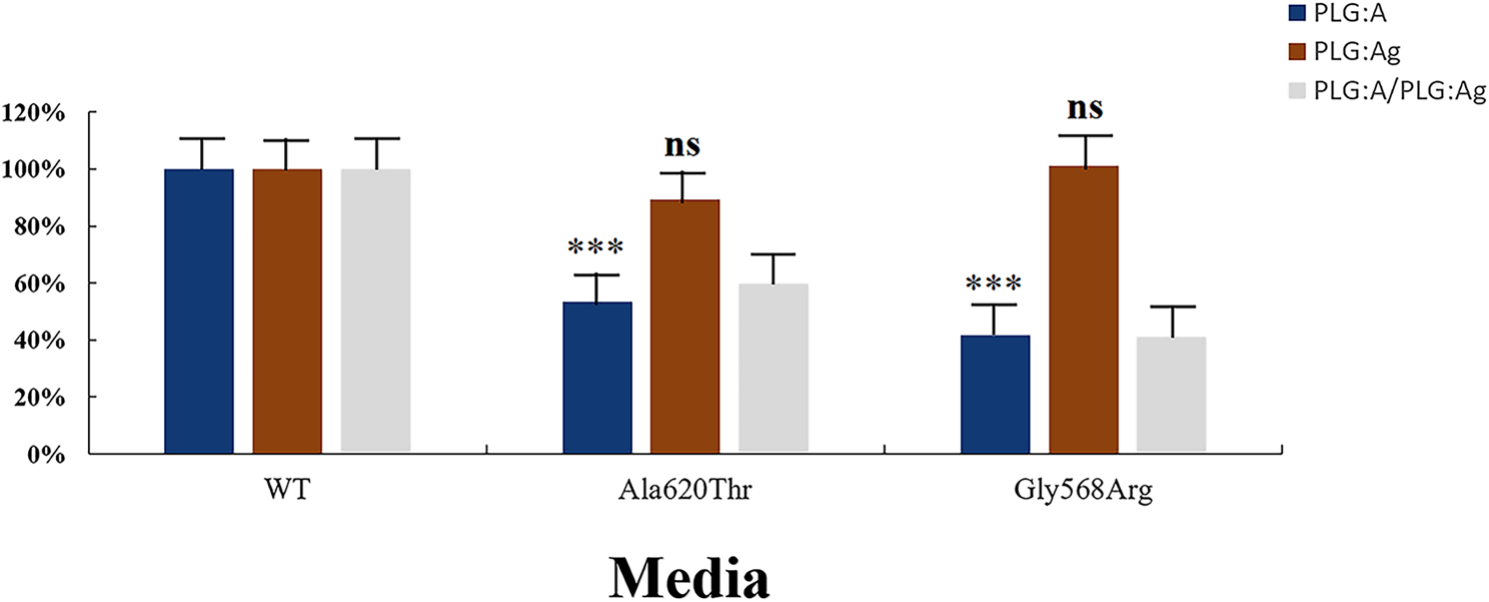
Quantitative analysis of PLG protein in the media was conducted by ELISA. (“ns” indicates no significant difference, *p* > 0.05; “***” indicates a significant difference, *p* < 0.001). PLG: A (brown bars); PLG: Ag (blue bars); and PLG: A/PLG: Ag (grey bars)

RT-qPCR analysis revealed no statistically significant differences in mRNA expression levels between PLG-WT and the mutations (Fig. 6). WB analysis demonstrated that the protein bands of PLG-A620T and PLG-G568R mutations in both cell lysates and media showed no significant differences compared to PLG-WT (Fig. 7). ELISA results indicated that in cell lysates, PLG: Ag levels were 88.7% ± 2.6% for PLG-A620T and 93.2% ± 1.8% for PLG-G568R; in media, the levels were 98.7% ± 4.9% for PLG-A620T and 101.2% ± 5.1% for PLG-G568R. The PLG: Ag content in the cell lysates or media of the two mutations was not statistically significantly different from that of PLG-WT. However, both mutations exhibited significantly reduced PLG: A/PLG: Ag ratios compared to PLG-WT, suggesting preserved secretory function but impaired enzymatic activity (Supplementary Fig. 2; Fig. 8).

## Discussion

Hereditary PLG deficiency can be classified into two subtypes: Type I (reduced activity and antigen levels) typically causes ligneous conjunctivitis, while Type II (reduced activity with normal antigen levels) predisposes to venous thromboembolism [11, 13]. We report a Type II case in a 60-year-old woman presenting with acute ischemic stroke, showing characteristic laboratory findings (27% activity with normal antigen levels). Genetic testing revealed two pathogenic PLG mutations (p.Gly568Arg and p.Ala620Thr) meeting ACMG criteria (PS1/PM2/PP3/PP4) [14], absent in 100 controls, with autosomal recessive disorder. This case demonstrates the classic Type II triad: selective activity deficiency, thrombotic predisposition, and confirmed pathogenic mutations.

The p.Ala620Thr mutation was first identified by N Aoki et al. in 1978 [15], whereas p.Gly568Arg is a novel pathogenic mutation. Both were predicted to be pathogenic by multiple bioinformatics tools and are located at highly evolutionarily conserved residues that are crucial for the structure and function of PLG. Structural modeling indicates p.Ala620Thr forms three additional hydrogen bonds with the catalytic triad (His603-Asp646-Ser741), potentially distorting the active site [16], whereas p.Gly568Arg adjacent to the t-PA/u-PA cleavage site (Arg561-Val562) creates steric hindrance through a new hydrogen bond with Leu686, impairing proteolytic activation [9, 17] The research by Chen et al. [17] demonstrated that the p.Ala620Thr mutation would interfere with the normal spatial conformation of the protein, forming structurally abnormal proteins and weakening the catalytic activity of plasminogen. This is consistent with the case and conclusion discussed in this article. This confirms that the mutation may significantly impair the catalytic activity of the protein, thereby leading to hereditary plasminogen deficiency.The compound heterozygous mutation status observed in the proband demonstrated a synergistic effect, as evidenced by a markedly reduced PLG: A level compared to that of heterozygous relatives carrying only a single mutation. This synergistic interaction resulted in severely impaired fibrinolytic activity, thereby significantly elevating the risk of thrombosis.

This study comprehensively characterized the molecular pathogenesis of type II PLG deficiency through parallel investigation of human mutation expression systems and existing transgenic mouse data [18]. Our in vitro experiments demonstrated that both PLG-A620T and PLG-G568R mutations maintained wild-type-level mRNA expression as shown by RT-qPCR, exhibited normal protein synthesis and secretion in WB and ELISA analyses, yet displayed significantly reduced enzymatic activity. These findings collectively establish a clear “conformational defect → functional impairment” pathogenic mechanism in human systems. Notably, this contrasts with the compensatory mRNA upregulation observed in PLG-A620T transgenic mice, a discrepancy we attribute to species-specific differences. The 22% non-conservative amino acid divergence between human and mouse PLG likely enhances mutation protein misfolding in murine endoplasmic reticulum (ER), triggering unfolded protein response-mediated mRNA upregulation. In contrast, human mutations achieve proper folding and secretion but retain catalytic defects. This comparative analysis highlights critical interspecies variation in protein quality control mechanisms and emphasizes the necessity of human-relevant models for translational research.

PLG deficiency presents ongoing clinical challenges, including heterogeneous diagnostic criteria and a lack of targeted therapies, with fresh frozen plasma remaining the primary treatment option in China due to the absence of approved PLG-specific replacement therapies [19, 20]. The condition’s thrombotic predisposition, likely mediated by impaired fibrinolytic capacity and defective clot clearance [21, 22], underscores the need for improved diagnostic strategies. Based on our findings, we recommend routine implementation of PLG genetic testing, especially for two key patient groups: those with family histories of hereditary PLG deficiency and individuals presenting with unexplained cerebral infarction. Establishing such screening protocols would enable early intervention to mitigate thrombotic risks.

## Conclusions

This study identifies a novel compound heterozygous PLG mutation (p.Ala620Thr/p.Gly568Arg) as the molecular basis for type II deficiency-associated cerebral infarction. Structural and functional analyses demonstrate these mutations specifically impair protein function through conformational disruption rather than affecting biosynthesis or secretion. Currently, research on PLG remains insufficient, particularly in elucidating the intrinsic mechanisms linking PLG dysfunction to thrombotic cerebrovascular events. This study’s exploration of these mechanisms offers potential biomarkers and interventional targets for early diagnosis and treatment strategies, while also further revealing potential associations between PLG dysfunction and thrombosis. However, the exact causative relationship between reduced PLG: A levels and cerebral ischemia has yet to be fully clarified, necessitating larger-scale and more systematic investigations to obtain more substantial supporting evidence.

## Supplementary Information

Below is the link to the electronic supplementary material.

Supplementary Material 1: Supplementary Fig. 1. TEG test results of the proband. (R: Reflects the overall activity of endogenous coagulation system factors; K: Mainly reflects the function of fibrinogen in the early stage of coagulation; Angle: Mainly reflects the overall function of fibrinogen in the coagulation process; MA: Mainly reflects the overall function of platelets (including aggregation, contraction and release, etc.); CI : Comprehensive index of coagulation function; EPL: Indicator for predicting fibrinolytic function; LY30: Indicator for measuring fibrinolytic function)

Supplementary Material 2: Supplementary Fig. 2. Quantitative analysis of PLG protein in transfected HEK293T cell lysates was performed by ELISA. (“ns” indicates no significant difference, *p* > 0.05)

Supplementary Material 3: Supplementary Tab 1. RT-qPCR primer sequences for PLG and GAPDH

## Data Availability

All data generated or analyzed during this study are included in this published article [and its supplementary information files].

## Abbreviations

PLG: Plasminogen
PLG:A: Plasminogen activity
PLG:Ag: PLG antigen
CSA: Chromogenic Substrate Assay
ELISA: Enzyme-linked immunosorbent assay
RT-qPCR: Quantitative real-time PCR
WB: Western blot
t-PA: Tissue-type plasminogen activator
u-PA: Urokinase-type plasminogen activator
ECM: Extracellular matrix
NTP: N-terminal peptide
SP: Serine protease
PC:A: Protein C activity
AT:A: Antithrombin III activity
PS:A: Protein S activity
PLG:Ag: Plasminogen antigen
PPP: Platelet-poor plasma
CLSI: Clinical and Laboratory Standards Institute
PCR: Polymerase chain reaction
TEG: Thromboelastography
ACMG: American College of Medical Genetics and Genomics
CDS: Coding sequence
HEK293T cells: Human embryonic kidney 293T cells
DMEM: Dulbecco’s Modified Eagle Medium
FBS: Fetal bovine serum
PMSF: Phenylmethylsulfonyl fluoride
SDS-PAGE: Sdium dodecyl sulfate-polyacrylamide gel electrophoresis
PVDF: Polyvinylidene fluoride
TBST: Tris-buffered saline with Tween-20
ECL: Enhanced chemiluminescence
MRI: Magnetic resonance imaging
ER: Endoplasmic reticulum
