# Supplementary figures and images for: Novel compound heterozygous mutations in plasminogen (p.Gly568Arg/p.Ala620Thr) impair protein structure and function in type II deficiency: mechanistic insights into a hereditary thrombogenic disorder
Source: Orphanet J Rare Dis. 2025 Dec 15;20:620. doi: 10.1186/s13023-025-04122-3 (PMC12706909; doi:10.1186/s13023-025-04122-3)

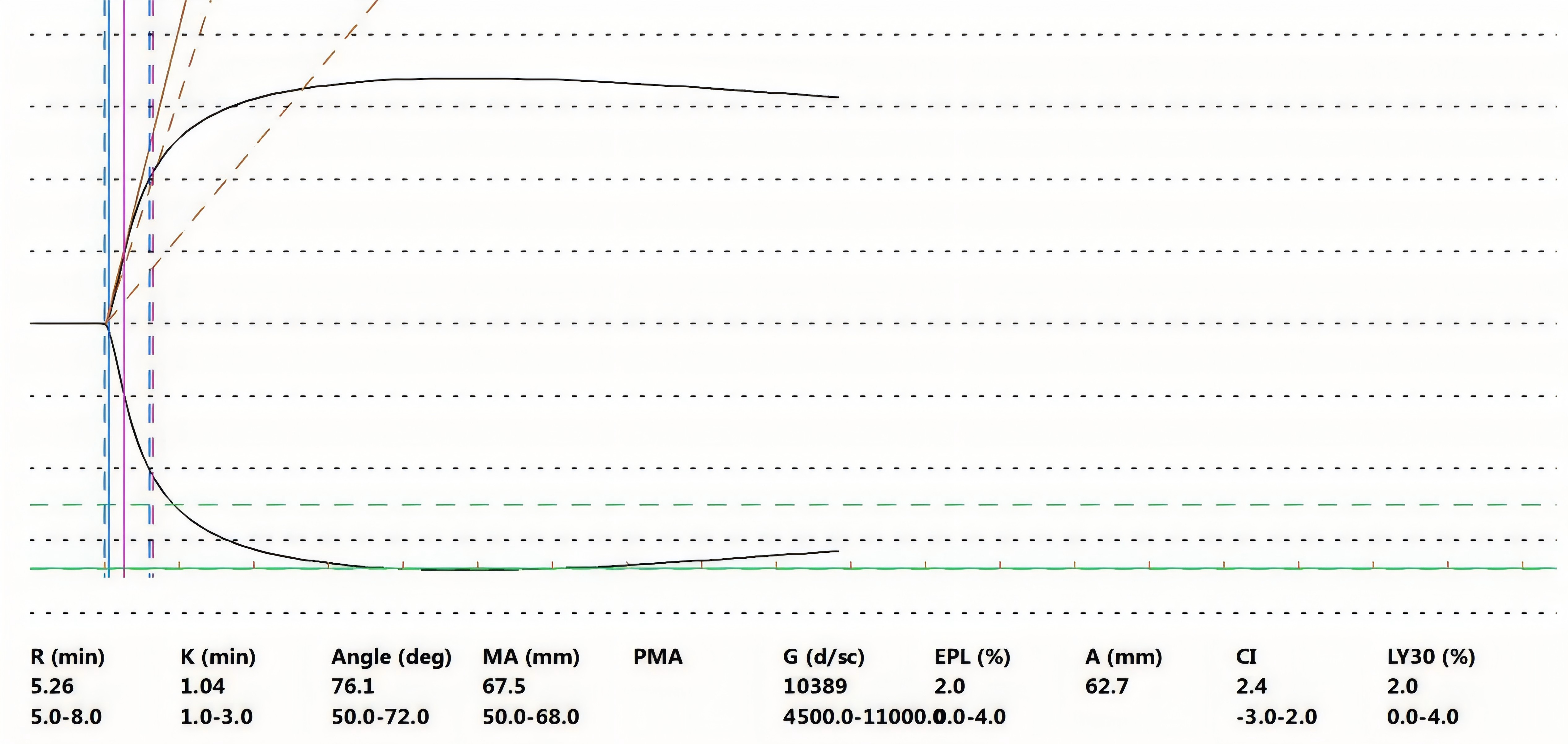

Supplement: Supplementary file 1 — Supplementary Material 1: Supplementary Fig. 1. TEG test results of the proband. (R: Reflects the overall activity of endogenous coagulation system factors; K: Mainly reflects the function of fibrinogen in the early stage of coagulation; Angle: Mainly reflects the overall function of fibrinogen in the coagulation process; MA: Mainly reflects the overall function of platelets (including aggregation, contraction and release, etc.); CI : Comprehensive index of coagulation function; EPL: Indicator for predicting fibrinolytic function; LY30: Indicator for measuring fibrinolytic function) [file 13023_2025_4122_MOESM1_ESM.png]

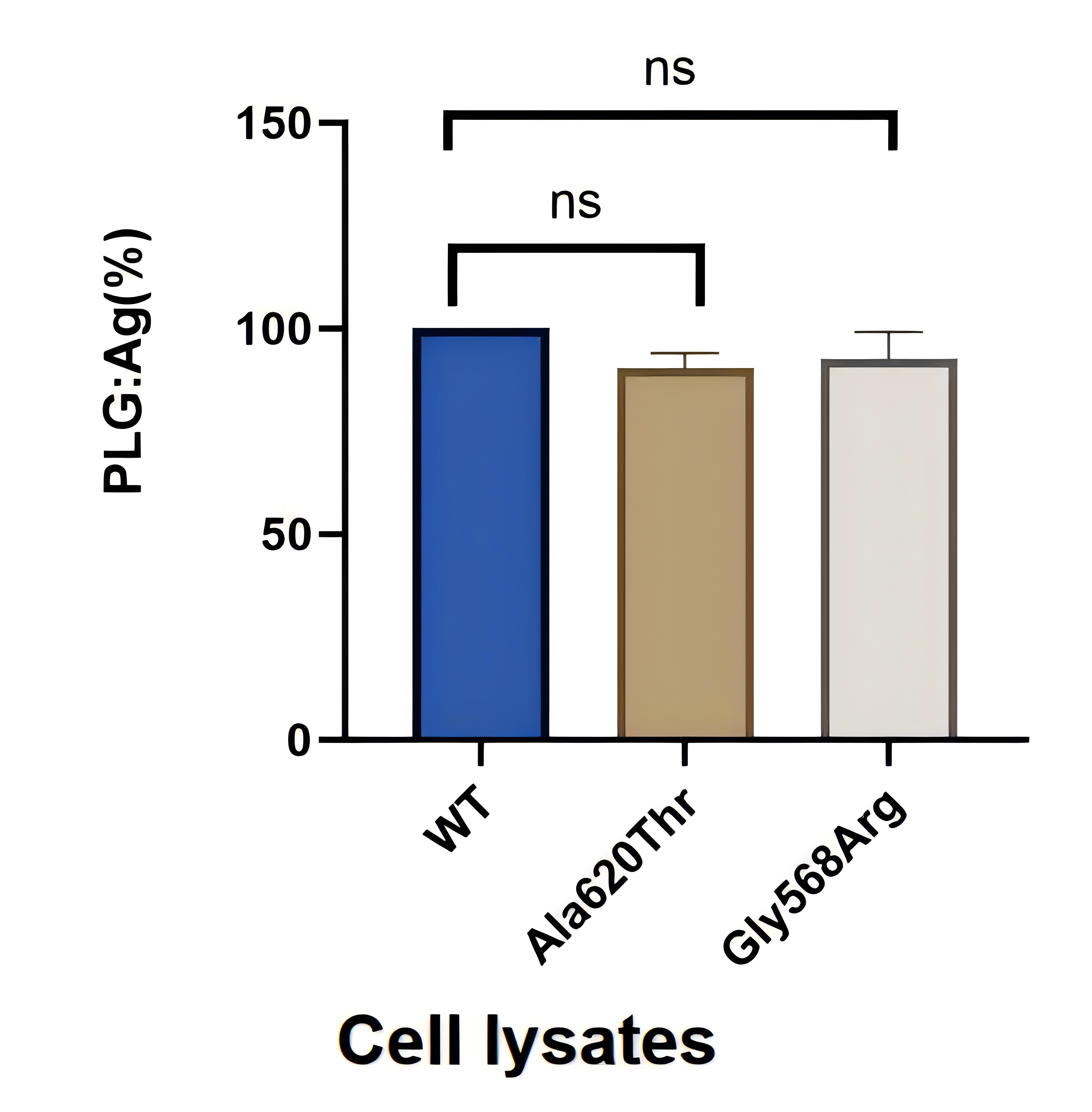

Supplement: Supplementary file 2 — Supplementary Material 2: Supplementary Fig. 2. Quantitative analysis of PLG protein in transfected HEK293T cell lysates was performed by ELISA. (“ns” indicates no significant difference, p > 0.05) [file 13023_2025_4122_MOESM2_ESM.png]
