# Supplementary material for: Novel compound heterozygous mutations in plasminogen (p.Gly568Arg/p.Ala620Thr) impair protein structure and function in type II deficiency: mechanistic insights into a hereditary thrombogenic disorder
Source: Orphanet J Rare Dis. 2025 Dec 15;20:620. doi: 10.1186/s13023-025-04122-3 (PMC12706909; doi:10.1186/s13023-025-04122-3)
Supplement: Supplementary file 3 — Supplementary Material 3: Supplementary Tab 1. RT-qPCR primer sequences for PLG and GAPDH [file 13023_2025_4122_MOESM3_ESM.docx]

**Table 1 RT-qPCR primer sequences for PLG and GAPDH**

| **Gene name** | **Forward sequences** | **Backward sequences** |
| --- | --- | --- |
| *PLG* | 5'-GCGACATTCTTGAGTGTGAA-3' | 5'-GGGGTTACGACAGTAATTCT-3' |
| *GAPDH* | 5'-AGAAGGCTGGGGCTCATTTG-3' | 5'-AGGGGCCATCCACAGTCTTC-3' |
